# A Case Report of Kratom-Induced Psychosis

**DOI:** 10.7759/cureus.16073

**Published:** 2021-06-30

**Authors:** Hunter A Cutlip, Ella Bushman, Lisa Thottumari, Raja Mogallapu, Michael Ang-Rabanes

**Affiliations:** 1 Psychiatry, West Virginia University School of Medicine, Martinsburg, USA

**Keywords:** substance-induced disorders, kratom, psychosis, substance recreational use, drug utilization review

## Abstract

This case report details a patient with a complex medical history who was brought for psychiatric evaluation. An abrupt switch in Kratom use patterns was identified as the most likely causative factor of his symptoms. Adrenal insufficiency and posttraumatic stress disorder (PTSD) were considered both in the differential and potential confounding factors in his presentation. This paper discusses current Kratom use trends in the United States, the drug's legal status, and the common reasons patients may use it. Additionally, research gaps regarding the safety and efficacy of Kratom's use for self-medication are addressed.

## Introduction

Kratom is a plant extract that has been used medicinally in Asia for over 150 years [1]. Patients who ingest Kratom report pain relief, increased energy, euphoria, and a sense of calm, dependent on dosage and personal factors [2]. Patients who use Kratom report improvements in chronic pain conditions, resolution of depression, and even reduced urge for opioid use [3]. However, the medicinal properties of Kratom are poorly studied and safety has not been sufficiently established [4]. Despite a lack of recommendation by any medical organization, not having a formal application for medical use per the FDA, and Kratom's status as a "drug of concern" by the Drug Enforcement Administration (DEA) [5], many patients still choose to self-medicate with Kratom as indicated by population surveys. In this case report, a patient presents with psychosis and insomnia considered secondary to acute Kratom intoxication. This paper seeks to bring attention to this potential mechanism for induction or exacerbating psychosis, and assess the currently available literature in order to consider the potential harms and benefits of Kratom use, as well as outline deficiencies that must be addressed prior to clinical acceptance of Kratom use.

## Case presentation

A 43-year-old white male with a history of posttraumatic stress disorder (PTSD), traumatic brain injury (TBI), and chronic primary adrenal insufficiency presented to the ED of a local Veterans Affairs (VA) hospital at approximately 0200 with visual and auditory hallucinations, as well as delusions of grandeur. He reported increased energy, and that he had not slept in seven days. The patient was noted to have poor self-care on admission. He was found to be confused and appeared to be paranoid. He endorsed impulsive decision-making over the past week. He also divulged a history of addiction to “pain relievers”. His initial vital signs were within normal limits. His presentation was suspicious for bipolar type I, which combined with severe agitation prompted the VA to give him haloperidol 5 mg and diphenhydramine 50 mg. He was then transferred from the VA to be admitted to an outside inpatient psychiatric unit. On arrival, the patient stated he was “too tired and cold” to participate in the intake interview. 

During the morning rounds, the patient was found to have a clear sensorium and demonstrated no evidence of delusions. The patient was more amenable to participating in an intake questionnaire. He reported an extensive military history, having served six tours of duty in the Army. During his service, he sustained several head injuries but did not report any symptoms related to this trauma. He had been diagnosed with PTSD by the VA after the military discharge6 but denied any symptoms at the time of admission. He had been treated for chronic primary adrenal insufficiency and depression through a primary care physician in the VA system in the past. He reported no prior inpatient or outpatient psychiatric treatment. He reported that he had been self-medicating for chronic low energy with Kratom over the past year. The treatment team confirmed that his presentation, exam, and rapid improvement did not justify imaging tests during this hospitalization.

The patient reported that his typical dose of Kratom was 6-8 pills in a day, on an “occasional” basis. He was unable to specify what “occasional” meant or what dose his pills contained. The patient states that he began experiencing increased feelings of loneliness and depression after his family had left on vacation. He decided to try liquid Kratom “for a change”. The patient then took an entire bottle of Kratom a week prior to his admission. He noted increased energy and was able to continue his daily activities without fatigue. As a result, he did not feel compelled to take his medications. He was not able to sleep at any point during the week. He began experiencing auditory and visual hallucinations five days after Kratom ingestion. He became concerned about the continued progression of his symptoms and presented to the VA ED. 

An assessment of the patient’s medical record yielded that six years prior to this admission he had presented to the VA for feelings of fatigue and depression. As a result, he was treated with Cymbalta 120 mg initially without improvement. Further laboratory testing resulted in a diagnosis of chronic adrenal insufficiency, which was determined to be the most likely contributing factor to his depression. He had been progressing through a titration off of the Cymbalta and had been taking 20 mg once a day on admission. Additionally, his adrenal insufficiency was addressed with hydrocortisone treatment consisting of 15 mg daily (10 mg in the morning and 5 mg at night). The work-up further diagnosed low testosterone, which was treated with 200 mg of testosterone weekly via intramuscular (IM) injections. Despite treatment, the patient had difficulty waking up in the morning and was prescribed 5 mg of Adderall daily. The patient reported that he relied on his wife for the appropriate and timely administration of his medications. 

The patient’s medications were restarted after rounds. After speaking with the patient, Kratom was considered the most likely cause of his acute psychosis. A urine drug screen taken at this time was negative for all tested substances. Labs for adrenocorticotropic hormone (ACTH), testosterone, follicle-stimulating hormone/luteinizing hormone (FSH/LH), and cortisol were ordered at that time to rule out adrenal insufficiency and steroid-induced psychosis, and the labs were completed the following day. Throughout the day, he was social with other patients and attended group therapy sessions. He was noted by staff who interacted with him as being wholly appropriate and aware of his condition. His mental status exam was grossly normal, and he denied experiencing depression, suicidal or homicidal ideation, and paranoia.

On the second day of his admission, the attending physician discussed with him the likelihood of Kratom being the root cause of his psychosis. Medication-induced psychosis was considered unlikely as his medication regimen had not been changed for several months prior to this episode, and the timeline provided by the patient indicated the onset of the psychosis prior to not taking his medications. Major depressive disorder was also considered, but onset at ingestion implies some role of Kratom and the patient did not have a history of psychosis associated with depression. The patient continued to improve and was discharged home with outpatient follow-up through the VA medical system. Follow-up efforts by the authors of this paper were unsuccessful.

## Discussion

In the United States, Kratom is available as an herbal supplement in the form of capsules, powder, or liquid, and is not prohibited by the Controlled Substances Act [5]. These products also lack oversight by the FDA, which contributes to a potential for contamination of Kratom-based products. Thus, safety and efficacy cannot be accurately assessed from current products on the market [2]. Use has been steadily increasing across the globe: the World Drug Report 2020 notes a significant rise in Kratom production as demonstrated by an exponential increase in the amount of the substance that was seized. Kratom has made up at least 50% of all “new psychoactive substances” seized worldwide since 2016, with a peak of nearly 90% in 2018 [6].

The primary active compounds in Kratom are mitragynine and 7-hydroxymitragynine (7-HMG) [7]. These act as agonists at mu opioid receptors. 7-HMG has a more analgesic effect than mitragynine and is thought to be more potent than morphine. 7-HMG is not present in all Kratom products, but those that do tend towards a maximum of 0.02%. Most Kratom products contain approximately 2% mitragynine. Mitragynine’s activity at opioid receptors accounts for its use as a substitute for opium or for mitigation of opioid withdrawal. The major chemicals associated with Kratom's effects were assessed for abuse potential in animal models by monitoring changes in the models' morphine intake. Mitragynine did not show evidence of abuse potential; however, 7-HMG demonstrates a clinically significant risk of abuse potential [1]. 

Kratom has a dose-dependent effect [2]. At low-to-moderate doses (1-5 g) of the raw leaves, it generally has a mild stimulant effect; at moderate-to-high doses (5-15 g), it is considered to have more sedative and relaxing effects. However, these effects are user-specific and the reaction may vary based on genetic factors, ingestion method, and use history [2]. Some acute adverse effects documented by users are anxiety, irritability, increased aggression, sedation, nausea, constipation, and itching. A retrospective study was conducted using the National Poison Data System and New York City Office of Chief Medical Examiner identifying Kratom exposures: in cases of Kratom toxicity, symptoms included agitation at 18.6%, tachycardia at 16.9%, drowsiness at 13.6%, vomiting at 11.2%, and confusion at 8.1%. More severe symptoms can also include seizure at 6.1%, hallucinations at 4.8%, respiratory depression at 2.8%, coma at 2.3%, and cardiac or respiratory arrest at 0.6% of cases [8].

Johns Hopkins University published a survey of 2,798 Kratom users in 2020 to determine real-world use patterns and perceived drug effects and benefits. Most patients used 1-3 g pills daily for depression, anxiety, or pain. Many of the respondents had chronic pain conditions, such as arthritis or muscle pain. These patients endorsed a strong belief in the effectiveness of Kratom for their conditions, with a mean efficacy rating for pain relief of 83.3 on a 100 point scale. Patients also report a significantly low rate of harmful or severe side effects and addiction. Additionally, of note was an opiate use reduction among users. A total of 40.9% of those responding to the survey reported using Kratom as a substitute or addiction treatment for chronic opiate use or abuse with a mean efficacy of 87.7%. Caution should be exercised in the extrapolation of this data in terms of drug safety and efficacy, due to the inherent bias towards safe use created by a survey of self-identified Kratom users. That said, these results are promising for the use of Kratom as an opioid alternative, and further research may provide significant benefit to those in whom opiate use is contraindicated or clinically inappropriate [3].

No clinical trials have been performed to date to establish the efficacy or safety of Kratom [2]. Additional concerns for extrapolation of the current data to humans have been established by studies performed with human plasma that have demonstrated significant differences from that of preclinical models that would be used to study these effects, such as with rats, mice, and dogs. Specifically, a 7-HMG metabolite called mitragynine pseudoindoxyl was discovered to be created in human plasma. This is an opiate with a higher potency than either mitragynine or 7-HMG [4]. Clinical trials would therefore be paramount in order to make any valid claims of safety for Kratom use. 

Currently, there is neither a specific dose that has been established to cause an overdose, nor is there any evidence of what predisposes a patient for psychosis as opposed to a seizure or a minimally responsive state [9]. A retrospective analysis of the State Unintentional Drug Overdose Reporting System (SUDORS) for Kratom overdoses reported that Kratom was found in 0.56% (152/27,338) of all overdose deaths reported to the system from 27 states between July 2016 and December 2017. Of these, Kratom was considered the cause of death in 91 cases. Of note, opiates and similar substances were present in a significant amount of cases. Up to 56% of cases positive for Kratom were found to be positive for fentanyl, as well. If this statistic is verified by further research into Kratom overdoses, the use of Kratom as an opiate abuse medication has significant safety concerns due to potential drug interactions of relapsing patients [10].

The patient described in this case report presented with a complex medical history. For this reason, it is necessary to delineate the effects of Kratom from other potential sources of psychosis. The timing of symptom onset implies the significance of substance or medication use in his psychosis, rather than an exacerbation of his underlying PTSD. It was therefore determined that his confusion and hallucinations originated from his extended period of insomnia. 

Steroid-induced psychosis was considered a significant alternative diagnosis. Patients using dexamethasone have been reported to present with psychosis which is manic-predominant. They experience auditory hallucinations, delusions of grandeur, and a feeling of having more energy. Patients can begin to act impulsively because of the feelings of elation associated with their improved energy, and additional symptoms can include insomnia and aggression [11,12]. Due to the short half-life of hydrocortisone and the timing of lab tests after admission, steroid-induced psychosis could not be diagnostically excluded. Patients diagnosed with steroid-induced psychosis become more sensitive to steroid-based medications and have been noted to experience a relapse of symptoms such as agitation after exposure to as little as 2.5 mg of prednisolone per day [13]. The long-term stability of this patient on his current dose illustrates the appropriateness of his regimen for treating his specific condition. This patient could only have been exposed to an excessive dose of steroids for a maximum of seven days. Steroid-induced psychosis has been reported to have a median onset of 11.5 days and only 39% of cases have an onset within the first week [14]. The present patient did not experience further symptoms after receiving home doses of his steroid medications. The combination of these factors should be considered sufficient to rule out steroid-induced psychosis in the presence of a more likely diagnosis. Further, the patient stated he stopped taking his chronic medications after feeling energized from the liquid Kratom and had drastically underestimated the effects of a different form of the drug.

## Conclusions

The psychiatric effects of Kratom are still poorly understood. We present a case of manic psychosis which is considered to be associated with Kratom. Such effects of Kratom should be considered as the drug becomes an increasingly popular way to self-medicate.
